# Global organization of phenylpropanoid and anthocyanin pathways revealed by proximity labeling of trans-cinnamic acid 4-hydroxylase in *Petunia inflata* petal protoplasts

**DOI:** 10.3389/fpls.2024.1295750

**Published:** 2024-09-19

**Authors:** Javiera Aravena-Calvo, Silas Busck-Mellor, Tomas Laursen

**Affiliations:** Section for Plant Biochemistry, Department of Plant and Environmental Sciences (PLEN), University of Copenhagen, Copenhagen, Denmark

**Keywords:** proximity labeling, phenylpropanoid pathway, anthocyanin pathway, *Petunia inflata*, protoplasts

## Abstract

The phenylpropanoid pathway is one of the main carbon sinks in plants, channeling phenylalanine towards thousands of products including monolignols, stilbenes, flavonoids and volatile compounds. The enzymatic steps involved in many of these pathways are well characterized, however the physical organization of these enzymes within the plant cell remains poorly understood. Proximity-dependent labeling allows untargeted determination of both direct and indirect protein interactions *in vivo*, and therefore stands as an attractive alternative to targeted binary assays for determining global protein-protein interaction networks. We used TurboID-based proximity labeling to study protein interaction networks of the core phenylpropanoid and anthocyanin pathways in petunia. To do so, we coupled the endoplasmic reticulum (ER) membrane anchored cytochrome P450 cinnamic acid 4-hydroxylase (C4H, CYP73A412) from *Petunia inflata* to TurboID and expressed it in protoplasts derived from anthocyanin-rich petunia petals. We identified multiple soluble enzymes from the late anthocyanin pathway among enriched proteins, along with other C4H isoforms, and other ER membrane anchored CYPs. Several of these interactions were subsequently confirmed by bimolecular fluorescence complementation (BiFC). Our results suggest that C4H co-localizes with enzymes from the phenylpropanoid- and downstream anthocyanin pathways, supporting the idea that C4H may serve as ER anchoring points for downstream metabolic pathways. Moreover, this study demonstrates the feasibility of using protoplasts to perform global mapping of protein network for enzymes in their native cellular environment.

## 1 Introduction

Phenylpropanoids are specialized metabolites present in land plants derived from l-phenylalanine. They are synthesized by the phenylpropanoid pathway, which branches out to produce several major compound classes such as monolignols, stilbenes, coumarins, volatile phenylpropanoids and flavonoids. Anthocyanins are flavonoid pigments that accumulate in different plant tissues and act as photoprotectants by absorbing UV light and scavenging free radicals (Guo et al., 2008). Additionally, these molecules provide a palette of colors ranging from orange to purple, pink and blue that serve to attract pollinators and seed dispersers to specialized plant tissues such as flowers and fruits (Winkel-Shirley, 2001). These showy attributes have made the phenylpropanoid pathway one of the best characterized pathways in plant specialized metabolism. The ability to guide phenylalanine towards specific downstream products on-demand, combined with experimental data from binary protein-protein interaction assays among some of the enzymes, has prompted the hypothesis that the pathway may organize into dynamic enzyme complexes termed metabolons (Knudsen et al., 2018; Laursen et al., 2015; Nakayama et al., 2019; Winkel, 2004). To organize the metabolons, enzymes of the cytochrome P450 superfamily have been suggested to serve anchoring points to the endoplasmic reticulum (ER) membrane for recruitment of soluble enzymes (Jørgensen et al., 2005; Laursen et al., 2021).

**Figure 1:**
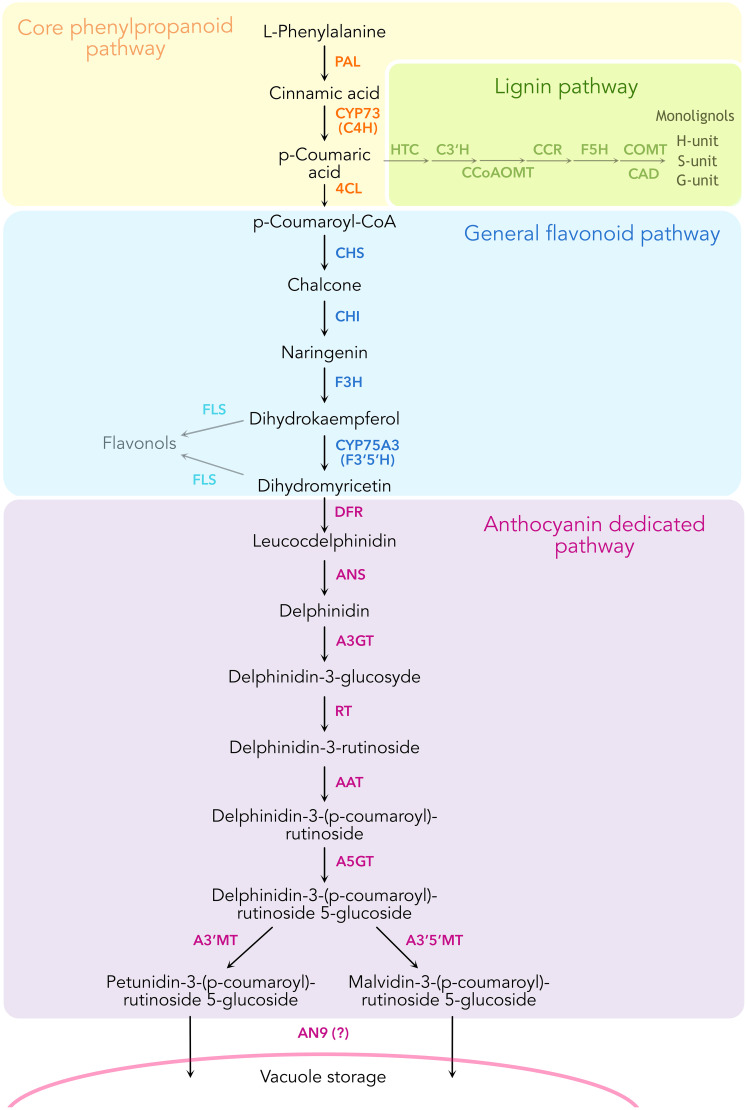
Schematic representation of the enzymatic reactions leading to the biosynthesis of anthocyanins in *Petunia inflata*, showing some of the branching pathways such as lignin and flavonol biosynthesis.

The general phenylpropanoid pathway (**Figure 1**) converts phenylalanine to 4-coumaroyl-CoA by the action of the sequential enzymes phenylalanine ammonia-lyase (PAL), ER membrane anchored P450, cinnamic acid 4-hydroxylase (C4H, CYP73A412), and the soluble 4-coumaryl-CoA ligase (4CL). This pathway remains one of the few that have been proven to form a metabolon involving substrate channeling and protein-protein interactions (Achnine et al., 2004; Rasmussen and Dixon, 1999). Substrate channeling has been demonstrated by isotopic dilution experiments in tobacco cell cultures and microsomes incubated with labeled l-phenylalanine and the intermediate trans-cinnamic acid. Here, [^3^H]-4-coumaric acid produced from feeding labeled [^3^H]- l -phenylalanine did not equilibrate with added unlabeled trans-cinnamic acid, revealing the rapid channeling of the latter intermediate from PAL to C4H (Rasmussen and Dixon, 1999). Subsequent work using fluorescence resonance energy transfer coupled with fluorescence lifetime imaging microscopy (FRET-FLIM) showed that tobacco C4H has a stronger affinity to interact with PAL1 compared to PAL2 (Achnine et al., 2004). Taken together, these findings support the formation of a metabolon in the early phenylpropanoid pathway, namely among PAL and C4H.

To date only a handful of metabolons have been experimentally proven (Laursen et al., 2016; Mucha et al., 2019; Zhang et al., 2018), largely due to the lack of proper techniques for elucidation of global protein interaction networks *in planta*. The recent development of proximity-dependent labeling has proven a powerful tool for the identification of protein networks in an untargeted manner that is gaining popularity also in the plant sciences (Xu et al., 2023). One of the most used approaches in plants is biotin-dependent proximity labeling, which is conducted by fusing a highly active biotin ligase to a protein of interest (bait) and expressed either stably or transiently in the organism. The addition of the biotin substrate allows the ligase to activate biotin enabling its covalent coupling to exposed lysine residues of all proteins in proximity to the bait. Biotinylated proteins are then captured by affinity purification, digested into peptides and identified by mass spectrometry (MS). One remarkable strength of proximity labeling is its ability to detect weak and transient interactions naturally happening in enzymatic complexes, which are frequently lost by other methods such as co-immunoprecipitation or tandem affinity purification. Furthermore, proximity labeling may provide spatiotemporal resolution of the protein interaction networks of a given biological process under native conditions by controlling labeling time (Branon et al., 2018; Gingras et al., 2019; Yang et al., 2021). Among the recently developed biotin ligases, TurboID stands out for its high efficiency at room temperature and fast labeling times, making it appropriate for studying dynamic interactions in plants (Arora et al., 2020; Mair et al., 2019; Zhang et al., 2019). TurboID has been applied to study protein interaction networks of regulators involved in NLR immune response in tobacco (Zhang et al., 2019), interactomes of endocytic TPLATE protein complex in Arabidopsis and tomato (Arora et al., 2020), effector protein networks in maize during infection with *Ustilago maydis* (Shi et al., 2023), among others. Moreover, it has allowed the elucidation of proteomes from cell-type specific compartments in Arabidopsis guard cells without the challenge of performing subcellular fractionation (Mair et al., 2019). In this study, we developed a TurboID-based proximity labeling approach to elucidate enzyme networks in the anthocyanin pathway. Through transient expression in protoplasts isolated from anthocyanin-rich Petunia inflata petals, which provide a fast and reliable transformation system, we demonstrate multiplexed proximity labeling experiments without the need to generate stable mutant plant lines. By using this system with C4H as bait fused to TurboID, we found enrichment of several cytosolic enzymes from the late anthocyanin pathway. Pair-wise protein interaction assays among the proximal candidate proteins and C4H by bimolecular fluorescence complementation (BiFC) resulted in several positive protein associations in the ER. Our results suggest that these enzymes co-localize on the ER surface near C4H, indicating the presence of higher order metabolic complexes in the anthocyanin pathway, which may be anchored by interactions with C4H. This is the first study reporting P450 protein networks elucidated by proximity labeling and stands as the basis for future experiments investigating metabolon formation in anthocyanin biosynthesis.

## 2 Material and Methods

### 2.1 Plant material and growing conditions

*Petunia inflata* seeds were obtained from the Amsterdam petunia germplasm collection (Strazzer et al., 2023) and germinated in pots under greenhouse conditions at 25°C day and 22°C night, with a photoperiod of 16h light/8h dark. For protoplast isolation, metabolomics and proteomics analysis, corolla tissue was collected from bud stage (S3) and freshly opened flowers (S5).

### 2.2 Protoplasts isolation

Isolation of protoplasts was performed as described in Faraco et al., 2011. P*. inflata* flower buds at stage 3 (S3) and freshly opened flowers at stage 5 (S5) were collected. The anthocyanin-rich epidermal layer was peeled from the petal and used for protoplast isolation. The tissue was incubated overnight in TEX buffer (Gamborg B5 medium, 500 mg/L MES, 750 mg/L CaCl_2_, [2*H_2_O] 250 mg/L NH_4_NO_3_, and 0.4 M sucrose, pH 5.7), containing 0.2% Macerozyme R10 and 0.4% Cellulase R10 (Duchefa). After 16 h, the protoplast suspension was filtered with a 100 µm cell strainer (Falcon) and washed three times with TEX buffer, with a 100 x g centrifugation step between washes. The protoplasts were counted using a hemocytometer and subsequently used for global proteomics and proximity labeling experiments.

### 2.3 Total protein isolation from protoplasts

A volume of isolated protoplasts containing 5 x 10^5^ cells was taken in three replicates, pelleted by centrifugation at 100 x g and lysed with 150 µL RIPA buffer (50 mM Tris, 150 mM NaCl, 1 mM EDTA, 1% NP40, 0.1% SDS, 0.5% sodium deoxycholate) supplemented with protease inhibitor (cOmplete™, Roche), 1 mM DTT and 1 mM PMSF. The lysate was centrifuged at 15000 x g at 4°C to remove cell debris and the supernatant was transferred to a new protein Lo-Bind tube. One hundred µg of the total protein extract were used for mass spectrometry analysis.

### 2.4 Sample preparation for global proteomics

Sample extracts were diluted with 1:1 with 2x SDS lysis buffer (10% SDS, 100 mM Tris pH 8.5), reduced with 5 mM tris(2-carboxyethyl)phosphine (TCEP) for 15 min at 55°C, and alkylated with 20 mM chloroacetamide for 30 min at room temperature. Protein purification and digest was performed following the PAC protocol (Batth et al., 2019). Peptides were acidified with TFA (final concentration 1%) and purified using SDB-RPS StageTips (Kulak et al., 2014).

### 2.5 LC-MS global proteomics

Peptides were separated on a 25 cm column Aurora Gen2, 1.7uM C18 stationary phase (IonOpticks) with either a Vanquish Neo HPLC system or an EASY‐nLC 1200 HPLC (Thermo Scientific) coupled via a captive-spray source to a timsTOF pro2 (Bruker Daltonics) mass spectrometer operated in DIA-PASEF mode (Meier et al., 2020). Peptides were loaded in buffer A (0.1% formic acid) and separated with a non-linear gradient of 2 – 35% buffer B (0.1% formic acid, 99.9% acetonitrile) at a flow rate of 400 nL/min over 90 min. Total run time was 101min including washing phase. The column temperature was kept at 50°C during all LC gradients.

### 2.6 Global proteomics analysis

For global proteomics of petunia protoplasts, data-independent acquisition (DIA) was processed in library-free mode using DIA-NN software (Demichev et al., 2022), using the *Petunia inflata* proteome (version 1.0.1, from https://solgenomics.net/) containing all proteins translated from the available genome (Bombarely et al., 2016). Search settings were as follows: Protease trypsin, peptide length 7-35 residues, precursor m/z 300-1800, precursor charge 1-4, maximum missed cleavages 1, Met oxidation as variable modification (up to 2 allowed), carbamidomethylation of Cys as fixed modification and N-termin Met excision enabled. Match-between-runs (MBR) was enabled, and Robust LC was used as quantification strategy. The R package DIAgui (https://github.com/mgerault/DIAgui) was used to calculate LFQ and iBAQ values from the intensities reported by DIA-NN. The iBAQ values were filtered to keep only proteins quantified in all three replicates in at least one group and subsequently, missing values were imputed using quantile regression imputation of left-censored data (QRILC) method (Zhang et al., 2018), assuming missing values not at random. Differential analysis of protein expression was done in R using the DEP package (Zhang et al., 2018). The mass spectrometry proteomics data have been deposited to the ProteomeXchange Consortium via the PRIDE (Perez-Riverol et al., 2022) partner repository with the dataset identifier PXD055749.

### 2.7 Construction of plasmids for proximity labeling assays

Full-length C4H (C4H412, Pinf101Scf00951g08008.1) was amplified from cDNA isolated from *P. inflata* corollas with the primers listed in **Supplementary Table 1**. The resulting PCR fragment was subsequently cloned in pJET and verified by sequencing. TurboID sequence was amplified from Addgene plasmid #127350 (Mair et al., 2019). To build the constructs for proximity labeling, TurboID was fused at the C terminus of C4H, including a 10 amino acid linker SGGGGGSGGG between the proteins. To generate C4H-TurboID-EGFP and C4H-TurboID fusions, the genes C4H, TurboID and EGFP were separately amplified by PCR using primers (**Supplementary Table 1**) that allowed 15-20 nucleotides overlap among the PCR fragments and the destination vector for a seamless fusion. To build the TurboID control for the ER compartment, the first 120 nucleotides corresponding to the ER transmembrane domain of *P. inflata* CYP51 were amplified by PCR. The PCR fragments were fused and cloned into a binary vector (pPZP200 backbone), after the CaMV35S promoter (p35S) by Gibson Assembly using NEBuilder^®^ HiFi DNA Assembly Master Mix (NEB). The resulting plasmids containing p35S::C4H-TurboID-EGFP, p35S::C4H-TurboID and p35S::ER-TurboID-EGFP were subsequently verified by sequencing.

### 2.8 Protoplast transformation

A total 1.4 x 10^6^ protoplast cells per sample were resuspended in MMM solution (0.5 M mannitol, 15 mM MgCl_2_, 0.1% MES). 20 µg of supercoiled plasmid was added to the protoplasts together with PEG solution (0.4 M mannitol, 0.1 M Ca(NO_3_)_2_, 40% polyethylene glycol (PEG 4000)) and incubated at room temperature for 1 h. Then, the protoplasts were washed three times with TEX buffer, resuspended in 2 mL TEX after the last centrifugation and incubated in darkness at 26°C for 16 h.

### 2.9 Microscopy/subcellular localization

Transformed protoplasts expressing C4H-TurboID-EGFP and ER-TurboID-EGFP were visualized by fluorescence microscopy (Nikon Eclipse Ni-U), 16h after transfection. GFP fluorescence was observed using excitation at 488 nm and emission at 514 nm.

### 2.10 Proximity labeling experiments

Three biological replicates of each transformation with p35S::C4H-TurboID, p35S::ER-TurboID-EGFP (subcellular compartment control) and with buffer (WT control, unstransformed) were incubated in 2 mL TEX buffer containing 50 µM biotin for 0 and 3 h at 25°C. Protoplasts were washed 5 times with ice-cold W5 solution (154 mM NaCl, 125 mM CaCl_2_, 5 mM KCl, 5 mM glucose, 2 mM MES, pH adjusted to 5.7), 100 x g centrifugation at 4°C between washes and pelleted after the last wash. To obtain total protein extracts, the protoplast pellet was lysed with ice-cold 200 µL RIPA buffer supplemented with proteinase inhibitor, 1 mM DTT and 1 mM phenylmethylsulfonyl fluoride (PMSF). The solution was incubated on ice for 15 min and then centrifuged for 10 min, 4°C at 15000 x g. The supernatant was transferred to a protein Lo-Bind microcentrifuge tube (Eppendorf) and total protein was subjected to colorimetric quantification with Pierce™ BCA protein assay kit (Thermo Scientific).

### 2.11 Western blot analysis

Total protein extract (10 µg) was separated by SDS-PAGE and transferred to PVDF membranes (BioRad). For detection of biotinylated proteins as result of TurboID activity, the membrane was blocked with 5% bovine serum albumin in PBS with 0.05% Tween-20 for 1 h and then incubated with HRP-conjugated streptavidin (1:20000, Thermo Fisher Scientific) with shaking for 1 h at room temperature or 4°C overnight. Blots were visualized using a GelDoc MP Imaging System (BioRad).

### 2.12 Affinity purification of biotinylated proteins

To capture biotinylated proteins, 100 µL slurry of magnetic beads conjugated with streptavidin (Thermo Fisher) was equilibrated by washing with RIPA buffer 3 times and subsequently incubated overnight with 100 µg of total protein extracted from petunia protoplasts, under continuous rotation at 4°C. After incubation, the beads were washed as follows: 3x with cold RIPA buffer supplemented with 1 mM DTT for 2 min, 1x with cold 1 M KCl, 1x with cold 0.1 M Na_2_CO_3_, 1x with 2 M urea in 10 mM Tris-HCl pH 8, 2x with cold RIPA buffer supplemented with 1 mM DTT and 3x with cold PBS to remove traces of detergents. One tenth of the beads was boiled in Laemmli buffer containing 20 mM DTT and 2 mM biotin and used for immunoblots. The rest of the beads were kept dried at -80°C until further processing.

### 2.13 On bead digest

Washed beads were incubated for 30 min with elution buffer 1 (2 M Urea, 50 mM Tris-HCl pH 7.5, 2 mM DTT, 20 µg/ml trypsin) followed by a second elution for 5 min with elution buffer 2 (2 M Urea, 50 mM Tris-HCl pH 7.5, 10 mM Chloroacetamide). Both eluates were combined and further incubated at room temperature over-night. Tryptic peptide mixtures were acidified to 1% TFA and loaded on Evotips (Evosep, Odense, Denmark).

### 2.14 LC-MS analysis of on bead digested samples

Peptides were separated on 15 cm, 150 μM ID columns packed with C18 beads (1.9 μm) (Pepsep) on an Evosep ONE HPLC applying the ‘30 samples per day’ method and injected via a CaptiveSpray source with a 10 μm emitter into a timsTOF pro mass spectrometer (Bruker) operated in PASEF mode (Meier et al., 2018). Briefly, the DDA-PASEF scan range for both MS and MS/MS was set to 100 - 1700 m/z, and TIMS mobility range to 0.6 – 1.6 (V cm^−2^). TIMS ramp and accumulation times were set to 100 ms each, and 10 PASEF ramps recorded for a total cycle time of 1.17sec. MS/MS target intensity and intensity threshold were set to 20.000 and 1.000, respectively. An exclusion list of 0.4 min for precursors within 0.015 m/z and 0.015 V cm^−2^ width was also activated.

### 2.15 Proximity labeling proteomics data analysis

Raw mass spectrometry data were analyzed with MaxQuant (v1.6.15.0) (Tyanova et al., 2016). Peak lists were searched against a manually curated version of the *Petunia inflata* FASTA database from Solgenomics.com, combined with 262 common contaminants by the integrated Andromeda search engine. False discovery rate was set to 1% for both peptides (minimum length of 7 amino acids) and proteins. “Match between runs” (MBR) was enabled with a Match time window of 0.7, and a Match ion mobility window of 0.05 min. The proteinGroups.txt file was loaded in R and the data matrix filtered to remove proteins “only identified by site”, “reverse” and “potential contaminants”. The filtered matrix was analyzed with R package DEP (Zhang et al., 2018). First, proteins were filtered to keep only proteins quantified in all three replicates in at least one (high stringency filtering) and subsequently missing values were imputed using MinProb. Differential enrichment of protein was comparing bait and controls at different timepoints, with manual adjustment of p-values using Benjamini-Hochberg (Benjamini and Hochberg, 1995). Significantly enriched proteins were obtained by an FDR of 0.05 and log_2_ fold change of 1.5. Volcano plots, PCA plots and iBAQ protein abundance ranks were plotted in RStudio (version 2023.06.0 + 421) and R (version 4.2.2), using proteomics datasets generated by DEP. The mass spectrometry proteomics data have been deposited to the ProteomeXchange Consortium via the PRIDE (Perez-Riverol et al., 2022) partner repository with the dataset identifier XXXXX.

### 2.16 BiFC assay

The genes C4Ha, C4Hb and AN9 were amplified from *P. inflata* cDNA using the primers listed in **Supplementary Table 1**. The coding regions of the genes C3’H, DFR, A3’MT and A3’5’MT were obtained from Twist Biosciences. The genes for bait proteins were subcloned into the pDEST-GW-VYNE by Gateway cloning, whereas genes for prey proteins were subcloned in pDEST-GW-VYCE by Gibson cloning (Gehl et al., 2009). For the positive control, the genes CYP71E2 and CYP79A1 from *Sorghum bicolor* (Laursen et al., 2016) were subcloned into the BiFC vectors. *Nicotiana benthamiana* leaves from 3-4 week old plants were infiltrated with combinations of *Agrobacterium tumefaciens* GV3101 (pMP90) strains containing the vectors for either nVenus, cVenus fusions, or ER marker (ER-mFRP) in a ratio 1:1:1, at a total OD_600_ of 0.4. Leaf discs were examined under confocal microscopy (Leica Stellaris 8, Leica Microsystems) 2-3 days after agroinfiltration. Venus complementation was detected using an excitation and emission wavelengths of 515 and 525-560 nm, respectively. The ER-mRFP signal was detected by excitation and emission wavelengths of 580 and 600-630 nm. Images were recorded sequentially, maintaining laser power and gain constant throughout all the samples.

## 3 Results

### 3.1 Global proteome profile of *Petunia inflata* petal protoplasts

To study spatial proximity of the enzymes in the phenylpropanoid pathway that might channel towards the biosynthesis of anthocyanins, we chose to use petunia flowers as a model system where this pathway has been extensively studied. Proximity labeling has been established in a few plant species, either by generating stable transgenic plants overexpressing bait-TurboID fusion constructs or by transient expression in leaves and leaf protoplasts (Arora et al., 2020; Mair et al., 2019; Zhang et al., 2019). Although agroinfiltration is possible in petunia petals for transient expression (Verweij et al., 2008), the tissue would not tolerate the successive rounds of infiltration, which are required to introduce biotin post transfection. To overcome this, we sought to develop a workflow for TurboID-based proximity labeling using protoplasts isolated from petal tissues.

**Figure 2:**
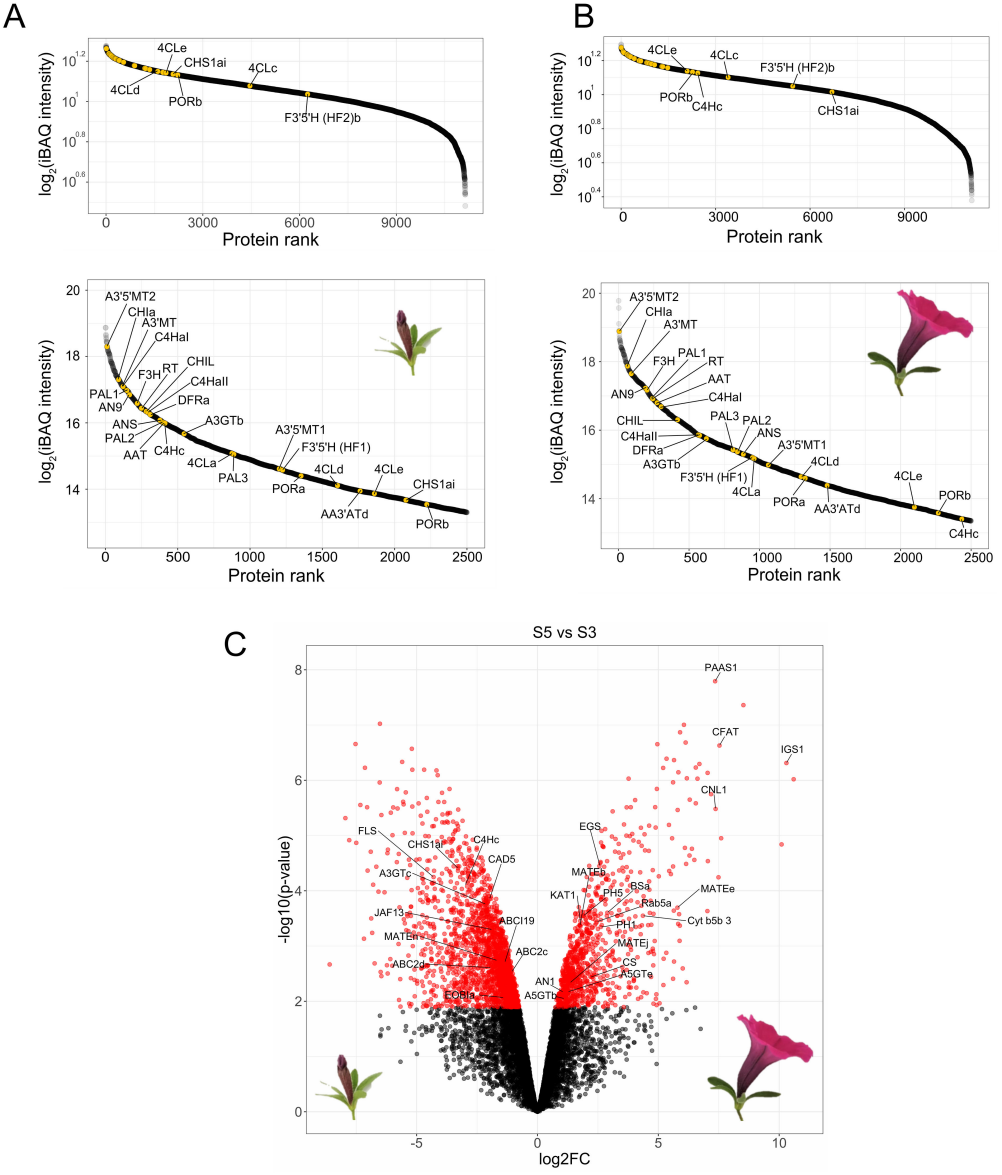
Global proteomics of protoplasts derived from *P. inflata* petals. Rank abundance curves for proteins quantified in S3 **(A)** and S5 **(B)**. Upper panels show to the ranking of the total number of proteins, whereas lower panel shows the 2500 most abundant proteins. Anthocyanin-related enzymes are indicated by yellow points. **(C)** Volcano plot of differentially enriched proteins in S5 *vs* S3. Red circles to the left correspond to significantly enriched proteins in S3, red circles in the right are proteins significantly enriched in S5. Log2FC, Log2 fold-change.

To assess the presence of the anthocyanin machinery in protoplasts isolated from mature flowers we performed global proteomics on the protoplasts isolated from mid-development flower buds (stage 3, “S3”) and from the epidermal layer of newly open flowers (stage 5, “S5”), as it is known that the gene expression of the enzymes (De Vetten et al., 1997; Quattrocchio et al., 1998) involved in the late biosynthesis of anthocyanins peak before flower opening. After filtering and imputation of the datasets, we obtained a total of 11,135 proteins quantified in both stages. Enzymes involved in the biosynthesis of anthocyanins were slightly more abundant in protoplasts from S3 petals compared to protoplasts from S5 petals, as seen in the ranked protein abundances for both stages (**Figures 2A, B**). Nevertheless, analysis of differential enrichment of proteins between S5 and S3 showed that abundances of most of the biosynthetic enzymes of the anthocyanin pathway were not significantly different in the two stages, with the exception of CHSa and a C4H paralog, C4Hc, which were significantly enriched in S3 (**Figure 2C**). Because anthocyanin biosynthetic enzymes are still among the most abundant proteins in S5 protoplasts, and flowers from this stage provided a much greater yield of protoplasts than flowers from S3, we proceeded to use S5 protoplasts for establishing proximity labeling experiments.

### 3.2 Development of proximity labeling in petunia protoplasts

**Figure 3:**
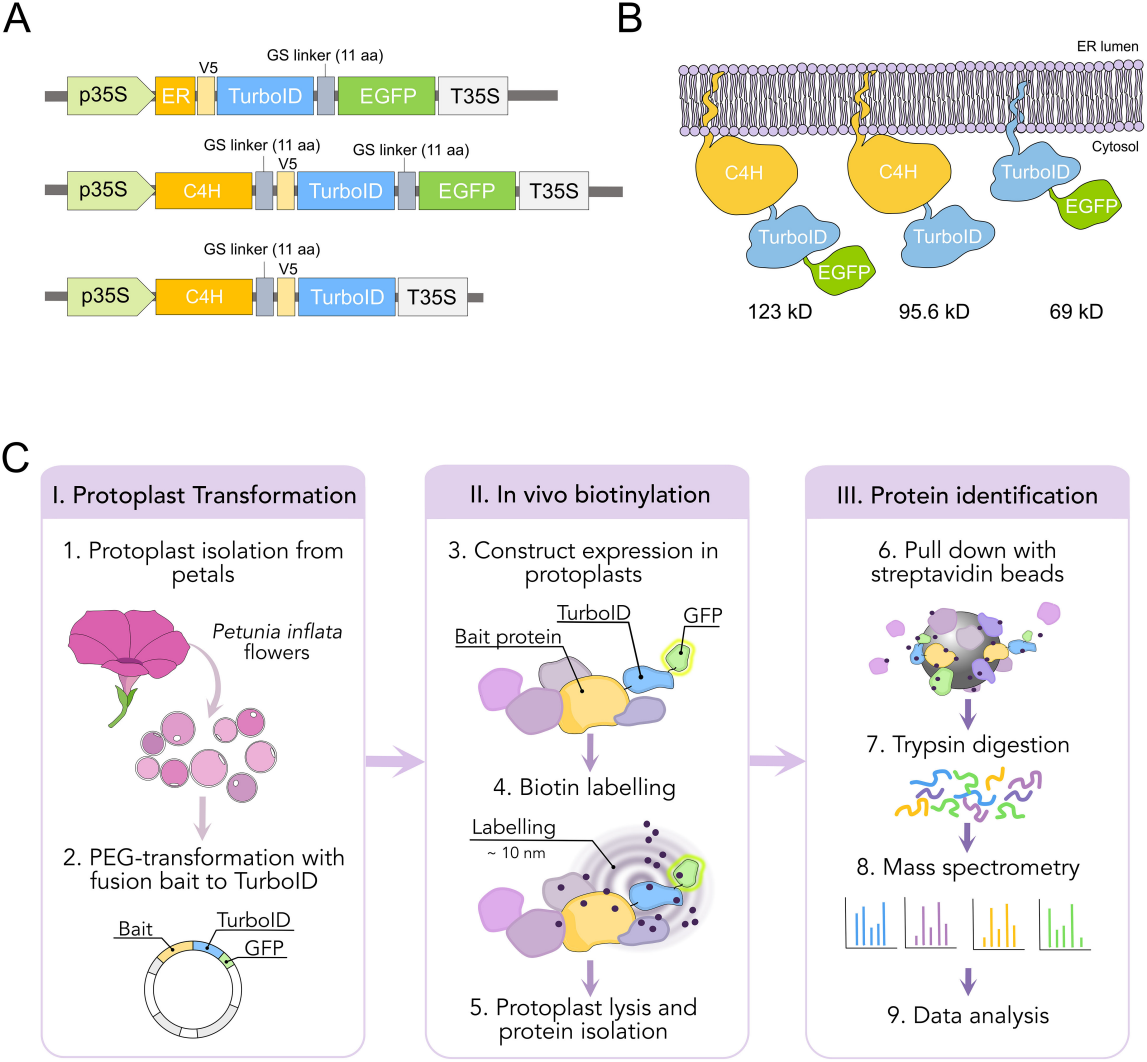
Design and workflow of proximity labeling experiments in protoplast from *P. inflata*. **(A)** Vector design for the genes encoding the fusion proteins ER-TurboID-EGFP, as ER control, and baits C4H-TurboID-EGFP and C4H-TurboID. All fusion genes under cauliflower mosaic virus 35S promoter **(B)** Diagrammatic representation of the fusion proteins associated to the ER membrane and their molecular weights. **(C)** Workflow of the proximity labelling procedure. I, protoplasts are isolated from flowers at anthesis and PEG-transformed with the plasmids pictured in **(A)** II, biotinylation is done *in vivo* by incubating the protoplasts with 50 µM biotin solution for 0 and 3 h, after which the protoplasts are washed three times and lysed. III, biotin-labelled proteins are affinity purified with streptavidin-conjugated beads for subsequent identification by mass spectrometry.

**Figure 4:**
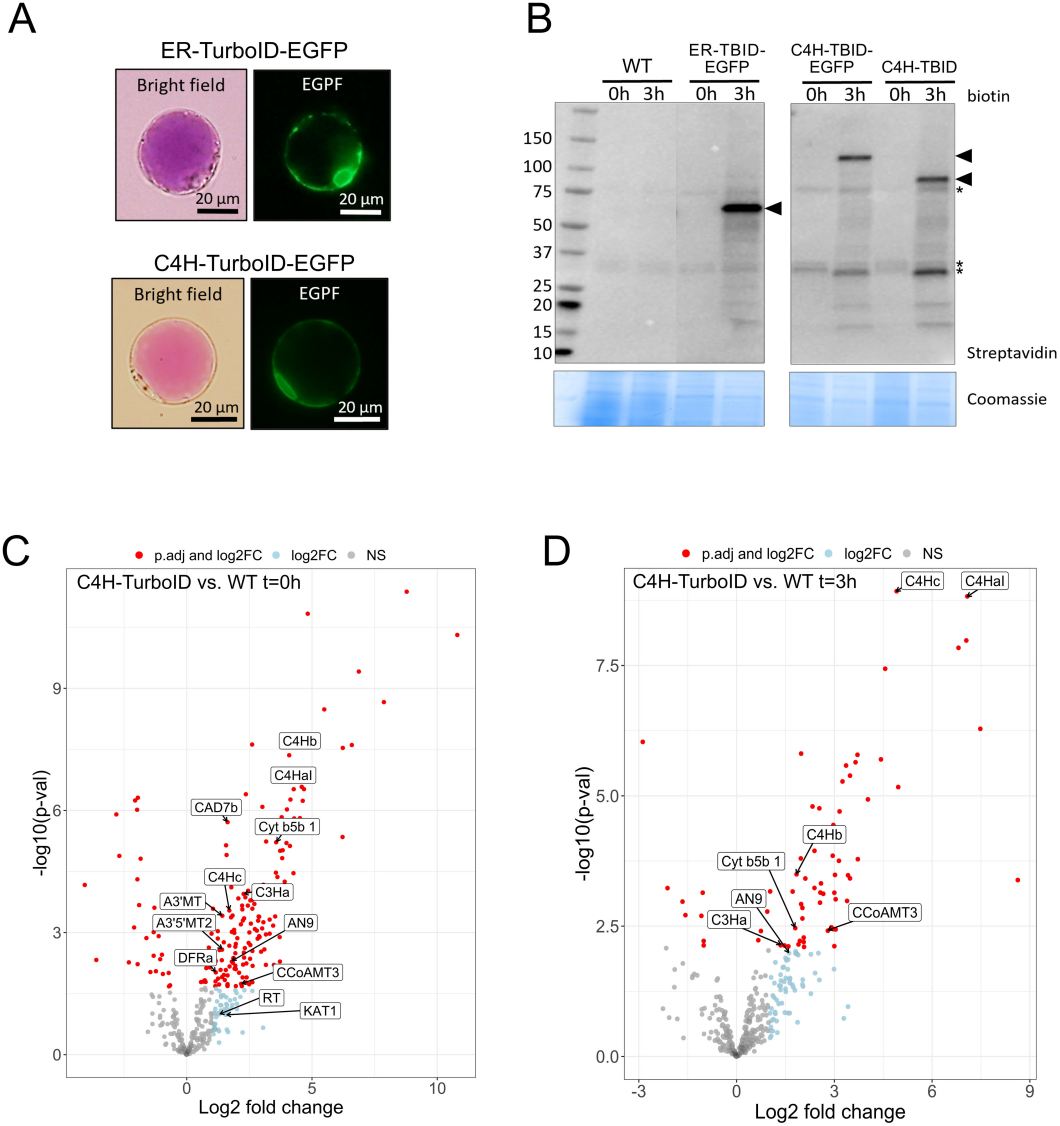
Establishment of proximity labeling in *P. inflata* petal protoplasts. **(A)** Subcellular localization of ER-TurboID-EGFP (upper panel) and C4H-TurboID-EGFP (lower panel) in protoplasts isolated from petal epidermis. **(B)** Expression and activity of ER-TurboID-EGFP, C4H-TurboID-EGFP and C4H-TurboID were analyzed by immunoblots using streptavidin-HRP antibody (upper panel). Protein extracts from WT (untransformed protoplasts) were loaded as negative control. Coomassie blue staining is showed as loading control. Points of arrow indicate the strongest bands corresponding to cis- biotinylation, ER-TurboID-EGFP: 69 kD; C4H-TurboID-EGFP: 123 kD; C4H-TurboID: 95.6 kD. Asterisks indicate endogenously biotinylated proteins **(C, D)** Volcano plots showing log2-fold change and –log10(p- value) values from differential expression analysis comparing C4H-TurboID *vs* WT (untransformed protplasts) at different biotin incubation timepoints: **(C)** t=0 hours and **(D)** t=3 hours. Significantly enriched proteins (adjusted p-value < 0.05) in C4H-TurboID are showed as red circles on the right half of each plot. Textboxes indicate the names of enzymes involved in the biosynthesis of anthocyanins: A3’MT, Anthocyanin 3’-methyltransferase; A3’5’MT2, Anthocyanin 3’5’-methyltransferase; AN9, glutathione transferase; C4HaI, C4Hb, C4Hc, C4H paralogs; C3Ha, cinnamate 3-hydroxylase a; CAD7, cinnamyl alcohol dehydrogenase 7a; CCoAMT3, Caffeoyl-CoA O-methyltransferase 3; Cyt b5b1, Cytochrome b5b 1; DFR, dihydroflavonol reductase; KAT1, 3-ketoacyl-CoA thiolase 1; RT, anthocyanin rhamnosyltransferase.

We performed TurboID-based proximity labeling in *P. inflata* petal protoplasts, following the steps shown in **Figure 3C**. Due to the vital importance of the N-terminal ER-transmembrane domain of eukaryotic P450s, TurboID was fused to the C-terminal of C4H by a short flexible linker (SGGGGGSGGG) between the proteins (**Figure 3A**). Petal protoplasts were transformed with vectors encoding for C4H fused to TurboID (C4H-TurboID) or with the addition of EGFP in the C-terminal of TurboID (C4H-TurboID-EGFP) for verifying the level of expression and subcellular localization of C4H fusions in the protoplast system. Additionally, another group of protoplasts was transformed with an expression vector encoding ER-TurboID-EGFP, consisting of TurboID fused N-terminally to the transmembrane anchor domain from the cytochrome P450 CYP51 and C-terminally to EGFP. This fusion represented a control for the ER labeling, having a subcellular localization similar to C4H (**Figures 3A, B**). Untransformed protoplasts (WT) were included as control for endogenously biotinylated proteins. Sixteen hours after transformation, the protoplasts were examined under epifluorescence microscope. Transformation efficiency of protoplasts ranged between 40-50% in all our experiments. We observed GFP fluorescence in protoplasts transformed with both ER-TurboID-EGFP and C4H-TurboID-EGFP fusion proteins, with fluorescence limited to membrane network structures surrounding the nucleus, characteristic for ER localized proteins (**Figure 4A**). These results indicate that the fusion proteins were successfully expressed and that the expressed proteins were correctly localized to the ER membrane.

To investigate the ability of TurboID fusions to biotinylate proteins in our protoplast system, we incubated transformed protoplasts with 50 µM biotin for 0 and 3 h. Next, we extracted proteins and analyzed the pattern of biotinylation by streptavidin immunoblot to evaluate the expression of catalytical active TurboID. Incubation with biotin for 3 h induced strong self-labeling (cis-biotinylation) in protoplasts expressing ER-TurboID-EGFP (69 kD), C4H-TurboID-EGFP (123 kD) and C4H-TurboID (95.6 kD) whereas only endogenously biotinylated proteins were observed in the absence of biotin (t =0) and the untransformed control (WT) (**Figure 4B**). Besides cis-biotinylation, trans-biotinylation was evident in protoplasts expressing all TurboID-fusions, visualized by a characteristic smear on the immunoblots (Arora et al., 2020; Mair et al., 2019), which corresponds to proteins labeled by encountering TurboID fusions inside the cell. To eliminate a possible interference of EGFP on C4H interactions, subsequent proximity labeling experiments were performed using the bait C4H-TurboID without EGFP.

### 3.3 Identification of proteins neighboring C4H

Biosynthesis of phenylpropanoids is expected to occur at the cytosolic surface of the ER membrane, where C4H has been proposed to mediate metabolon formation by coordinating assembly of soluble enzymes for production of phenylpropanoid derivatives (Winkel-Shirley, 1999). To investigate interaction networks around C4H, we affinity-purified the biotinylated proteins resulting after incubation with biotin. The amount of biotin-tagged proteins obtained was highly variable among the different treatments and barely detectable by streptavidin immunoblots (**Supplementary Figure 1A**), but were sufficient to quantify peptides by mass spectrometry. Label-free quantification (LFQ) identified 413 proteins after removing proteins with too many missing values. Principal component analysis (PCA) (**Supplementary Figure 2**) showed a clear separation between C4H-TurboID and control groups. ER-TurboID-EGFP grouped with WT controls at both timepoints, suggesting that the protein compositions and quantities of these groups were very similar. The proteomic data set from C4H-TurboID was first contrasted against WT to identify enriched proteins (possible interactors) and remove background proteins consisting of naturally biotinylated proteins such as Acetyl-CoA carboxylases (ACCases), and biotin carboxyl carrier protein (BCCP) subunits, as well as proteins binding non-specifically to the beads. Pair-wise comparison of C4H-TurboID and ER-TurboID-EGFP did not result in further enriched proteins, consistent with the clustering of WT and ER-TurboID-EGFP seen in the PCA (**Supplementary Figures 1**, **3**), suggesting that both control groups have a similar protein profile in this experiment.

After pair-wise comparison of the bait with WT samples, we ended up with 185 significantly enriched proteins in protoplast samples before biotin incubation (t= 0) and 69 proteins in the biotin treatment (t=3h) (**Figures 4C, D**). Most of the proteins identified were annotated for processes associated to mitochondrial metabolism, translation machinery and chaperones (**Supplementary Tables 2**, **3**). However, among the significantly enriched proteins we found additionally two C4H isoforms (C4Hb, C4Hc) and soluble enzymes from different branches of the phenylpropanoid pathway including the monolignol and anthocyanin branches. From the anthocyanin pathway, we identified dihydroflavonol synthase (DFR), anthocyanin rhamnosyltransferase (RT), the anthocyanidin 3’ and anthocyanidin 3’5’ O-methyltransferases (A3’MT and A3’5’MT) that perform the final methylation of delphinidin to form the anthocyanins petunidin and malvidin predominant in petunia (Provenzano et al., 2014), and a glutathione transferase (AN9), which has been suggested to be involved in the transport of anthocyanins to the vacuole (Alfenito et al., 1998; Buhrman et al., 2022; Mueller et al., 2000; Eichenberger et al., 2023). From the monolignol pathway, we found the soluble enzymes cinnamyl alcohol dehydrogenase (CAD), caffeoyl-CoA O-methyltransferase (CCoAOMT), and the ER membrane anchored *p*-coumarate 3-hydroxylase (C3’H, CYP98A2).

### 3.4 Validation of protein interaction in planta

**Figure 5:**
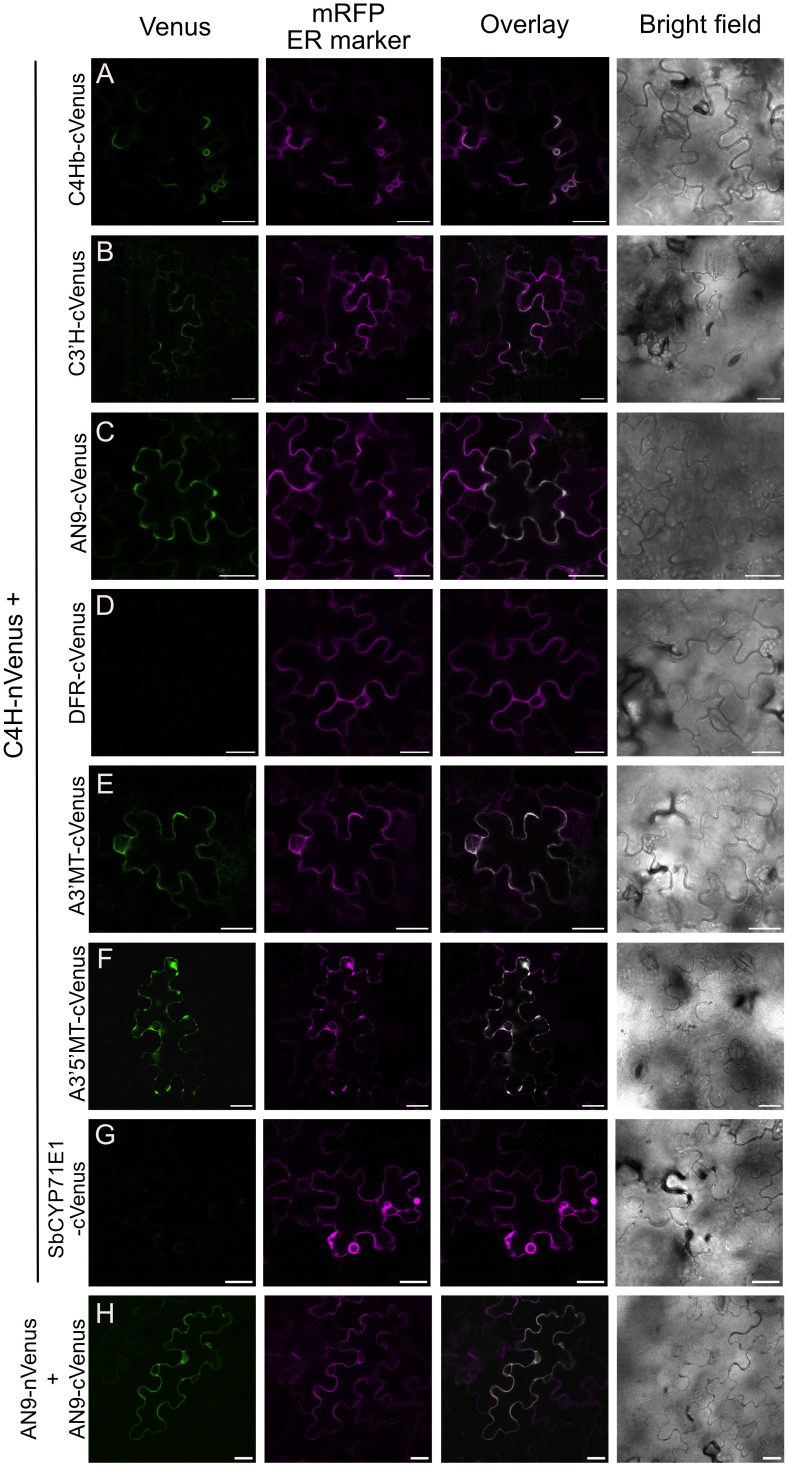
Bimolecular fluorescence complementation assays (BiFC) between C4H and enzymes of the anthocyanin pathway. Agrobacterium co-expressed in *N. benthamiana* leaves Complementation of Venus was detected 3 days after infection, by confocal microscopy. **(A-F)** BiFC pair-wise comparison between C4H-nVenus and cVenus fusions of C4Hb, C3’H, AN9, DFR, A3’MT and A3’5’MT, respectively. Scale bar = 25 µm. **(G)** Co-expression of C4H and CYP71E1 from *Sorghum bicolor*, as negative control for the BiFC assay. **(H)** Homodimerization of AN9 as positive control for BiFC assays.

To further validate the spatial proximity between C4H and the protein hits obtained in our proximity labeling assays, we performed bimolecular fluorescence complementation (BiFC) assays, adopting a method previously used in plants (Gehl et al., 2009). We fused an N-terminal fragment of yellow fluorescent protein Venus (nVenus) to the C terminus of C4H (C4H-nVenus) as bait and a C-terminal fragment (cVenus) to different prey proteins from the flavonoid and anthocyanin pathways that were enriched in our proximity labeling proteomics dataset (C4Hb, C3’H, AN9, DFR, A3’MT and A3’5’MT). Constructs were co-transformed in a pairwise manner into *N. benthamiana* leaves and fluorescence complementation was evaluated after 48-72 hours by confocal microscopy. We used AN9-nVenus together with AN9-cVenus as a positive control (**Figure 5H**), as this protein is known to form homodimers (Dixon et al., 2009; Mueller et al., 2000; Sun et al., 2012). We observed that co-expression of C4H-nVenus with the other P450s C4Hb-cVenus and C3’H-cVenus resulted in faint fluorescence localized in secluded regions of the ER (**Figures 5A, B**). Co-expression of C4H-nVenus with cVenus fused to the enzymes AN9, A3’MT and A3’5’MT resulted in fluorescence signals of variable strength co-localized with the ER marker mRFP (**Figures 5C, E, F**). On the other hand, we could not detect any Venus signal upon co-infiltration of C4H-nVenus with either DFR or the negative control *Sorghum* CYP71E1 (**Figures 5D, G**).

Further evaluation of the subcellular localization of Venus complementation derived from interaction between C4H with the soluble enzymes AN9, A3’MT or A3’5’MT revealed a recruitment to the ER membrane and a polarized distribution within the ER network towards the “edges” of the mesophyll puzzle shapes (**Figures 5C, E, F**). Interaction of C4H with A3’5’MT specifically resulted in fluorescence signal present in the ER, but not in the area surrounding the nucleus (**Figure 5F**, **Supplementary Figure 4**).

## 4 Discussion

### 4.1 *P. inflata* petal protoplasts provide a versatile system for proximity labeling experiments

Proximity labeling has proven to be a powerful tool to elucidate subcellular proteomes and protein-protein interaction networks (Xu et al., 2023; Yang et al., 2021). It stands out from traditional binary protein-protein interaction methods, such as yeast two-hybrid, bi-molecular fluorescence complementation (BiFC) and FRET methods, in providing an untargeted approach for studying protein-protein interactions in native conditions and can be used to detect weak or transient interactions that may be lost by other untargeted methods such as co-immunoprecipitation (Branon et al., 2018). To date, only a handful of studies have successfully reported biotin ligase-based proximity labeling in model plant species such as tobacco, *Arabidopsis thaliana* and tomato (Arora et al., 2020; Feng et al., 2023; Gryffroy et al., 2023; Huang et al., 2020; Khan et al., 2018; Lin et al., 2017; Mair et al., 2019; Zhang et al., 2019), mostly involving stable transformation of plants to express the bait of interest. Here, we demonstrate proximity labeling in *P. inflata*, which is an important model for anthocyanin biosynthesis and one of the few petunias with a sequenced genome (Bombarely et al., 2016). In addition, we provide a protocol for studying protein interactions in the biosynthesis of anthocyanins by transient expression in protoplasts from highly specialized cell types as the flower petals. Protoplasts have been extensively used to study biological processes such as light responses, signal transduction, protein interactions, and multi-omics (Sheen, 2001; Xu et al., 2022). Faraco et al. (2011) developed a protocol for isolation and transformation of petunia petal protoplasts with high transformation efficiencies (up to 60%), which enabled the study of subcellular processes related to anthocyanin accumulation and vacuole acidification (Faraco et al., 2017; Li et al., 2021). Here, we show that transient expression in petunia protoplasts makes a versatile alternative to stable transformation for performing proximity labeling assays, with a fast experimental turnaround.

Previous studies on temporal expression of biosynthetic genes from the anthocyanin pathway during flower development has revealed that anthocyanin biosynthesis peaks at stage 3 and decrease towards the end of the bud development and flower opening (Quattrocchio et al., 1993, 1999). Therefore, we were concerned that the anthocyanin pathway would already be downregulated in S5 flowers, which was used for preparation of protoplasts followed by proximity labeling. However, while our global proteomics analysis showed that enzymes belonging to the phenylpropanoid and anthocyanin pathways are among the most abundant proteins in protoplasts from stage S3 (**Figure 2A**), the biosynthetic enzymes remained highly abundant in protoplasts from open flowers (S5) (**Figure 2B**), similar as reported by (Prinsi et al., 2016). These findings contrast with previous studies based on transcript levels (Quattrocchio et al., 1993, 1999). Such discrepancies between gene expression measured at the transcript versus protein levels have previously been reported in plant systems (Mergner and Kuster, 2022), indicating that direct measurement of protein levels by mass spectrometry is preferable to determine the optimal tissues to perform proximity labeling experiments.

### 4.2 Considerations for proximity labeling experiments in *P. inflata* petal protoplasts

Plant protoplasts are cells where the cell wall has been removed and therefore can be comparable to mammalian cells due to the similar exposed cellular membrane (Sheen, 2001). Consequently, we chose concentrations of biotin (50 µM) typically used for proximity labeling experiments in HEK 293T cells (Branon et al., 2018) and in rice protoplasts (Lin et al., 2017). Our data suggests that endogenous biotin levels (t=0) provide sufficiently reliable TurboID-mediated biotinylation (**Figures 4C, D**), even though biotinylation was undetectable in immunoblots. We speculate that relying solely on labeling with endogenous biotin may reduce background and lead to more accurate identification of interactors, though this hypothesis remains to be systematically assessed. Two of the most challenging aspects of working with protoplasts relates to transformation efficiency and protein yields. Hence, approaches that obviate the requirement for biotin treatments would significantly decrease experimental complexity by reducing sample number and handling, and enable higher throughput in large-scale proximity labeling experiments. The main drawback of this approach is that the longer labeling times required could reduce temporal resolution, and so may not be suitable for experiments to determine interactions on short time scales. Nevertheless, our protoplast system is flexible and would allow users to choose short labeling times by adding biotin, or longer labeling without the addition of it, depending on the experimental question.

### 4.3 A glimpse into the C4H interactome

Protein-protein interactions among enzymes in the flavonoid pathway have been investigated in several plant species such as rice, *Arabidopsis*, soybean, snapdragon and torenia (Fujino et al., 2018; Nakayama et al., 2019), and these studies clearly demonstrate that biosynthesis of flavonoids involves formation of different enzyme complexes. Nevertheless, the exact functional consequences of such protein complexes are not fully understood, and their exact compositions do not seem to be conserved between different species (Nakayama et al., 2019).

C4H catalyzes the conversion of cinnamic acid to *p*-coumaric acid, an essential early step in the phenylpropanoid pathway (**Figure 1**) (Vogt, 2010). Because *p-*coumaric acid is a key branch point metabolite serving as substrate for production of coumarins, monolignols, stilbenoids, curcuminoids, floral volatiles and flavonoids, we speculate that C4H may play a role in controlling which downstream pathways are active through interaction networks with dedicated enzymes from individual metabolic branches. Using C4H as a bait has revealed several potential interactors from the routes branching out the general phenylpropanoid pathway, essentially downstream enzymes from the lignin and anthocyanin pathways, supporting the possible role of P450s as hub for metabolic complexes (Jørgensen et al., 2005; Laursen et al., 2015; Ralston and Yu, 2006; Zhang and Fernie, 2021).

Validation of selected protein hits by BiFC showed that C4H is in close proximity to enzymes which are several biosynthetic steps downstream in the anthocyanin pathway, such as AN9, A3’MT and A3’5’MT (**Figure 5**). The complementation of Venus fluorophore in the ER indicates that C4H recruits these soluble enzymes to the ER, reinforcing the hypothesis that P450s act as hotspots for metabolic complexes (Jensen et al., 2021; Jørgensen et al., 2005; Knudsen et al., 2018; Laursen et al., 2021). Our approach does provide novel insights on the subcellular localization of the individual biosynthetic steps for production of anthocyanins in *Petunia inflata*. Based on these results, we conclude that the biosynthetic machinery for production of anthocyanins including the final methylation step is localized in the cytosolic face of the ER membrane.

Additionally, our results indicate new associations between C4H and enzymes from the late steps of the biosynthesis of anthocyanin (A3’MT, A3’5’MT and AN9) in petunia and points to a complex organization of enzymes in the anthocyanin pathway. We propose that C4H acts as a hub in the ER for enzymes of the pathway and that these are organized in a complex where the structural enzymes from downstream reactions are in spatial proximity to the enzymes of the early steps, in association to the ER membrane (Burbulis and Winkel-Shirley, 1999; Winkel-Shirley, 1999).

Previous studies have proposed that C4H and the initial enzyme of the lignin pathway, C3’H, interact in the ER membrane in *Arabidopsis* and poplar epidermal cells (Bassard et al., 2012; Chen et al., 2011) by means of FRET and BiFC, respectively. Recently, a combination of yeast two hybrid (Y2H) and BiFC experiments suggested that C4H, C3’H (CYP98A3) and F5H (CYP84A1) are in close vicinity and co-localize in the ER membrane of *Arabidopsis* cells, but do not interact directly (Gou et al., 2018). So far our BiFC results appears to support interactions between C4H and C3’H, however to our knowledge, our results are the first to suggest interactions between C4H and enzymes of the anthocyanin pathway.

Surprisingly, none of the previously known interactors of C4H were identified among the enriched proteins in our proximity labeling experiment, such as phenylalanine ammonia lyase (PAL), P450 oxidoreductase (POR), membrane steroid binding protein (MSBP) and 4-coumarate-CoA ligase (4CL) (Achnine et al., 2004; Bassard et al., 2012; Gou et al., 2018). We cannot exclude that these and other proteins were not detected in our proximity labeling experiments due to intrinsic biases that could hinder labeling such as protein spatial conformation outside TurboID labeling radius, few or masked lysine residues on the surface, protein fold, size and abundance (Mair and Bergmann, 2022). It is important to note that also the protein interaction studies mentioned above, have been done in different plant species and tissues, so the interactions proposed cannot be generalized. We propose that future combinatorial investigations using proximity labelling with other enzymes of the anthocyanin pathway as baits would be a viable approach to achieve a holistic view of the organization of the anthocyanin pathway.

## 5 Conclusion

We demonstrate the use of proximity labeling in *P. inflata* protoplasts isolated from petals of open (S5) flowers. Using C4H as bait fused to TurboID, we identify enzymes from different branches of the phenylpropanoid pathway, supporting a central role of this enzyme as anchoring point for channeling of phenylalanine towards production of diverse compounds according to the specific needs of the plant cell.

This study demonstrates that protoplasts can be used to map protein networks in their native cellular environment using TurboID-based proximity labeling. This provides a new method to use proximity labeling to study protein interaction networks in specialized cell types, and may provide a blueprint for its use in plants that are otherwise recalcitrant to *Agrobacterium-*based transformation.

## Data Availability

The data presented in this study are deposited to the ProteomeXchange repository under accession numbers PXD055749 (Petal protoplast global proteomics) and PXD055778 (Proximity labeling).
