# Supplementary material for: Global organization of phenylpropanoid and anthocyanin pathways revealed by proximity labeling of trans-cinnamic acid 4-hydroxylase in Petunia inflata petal protoplasts
Source: Front Plant Sci. 2024 Sep 19;15:1295750. doi: 10.3389/fpls.2024.1295750 (PMC11446795; doi:10.3389/fpls.2024.1295750)
Supplement: Supplementary file 1 [file DataSheet1.docx]

Supplementary Material

Global organization of phenylpropanoid and anthocyanin pathways revealed by proximity labeling of trans-cinnamic acid 4-hydroxylase in *Petunia inflata* petal protoplasts

Javiera Aravena-Calvo^1^, Silas Busck-Mellor^1^, Tomas Laursen^1,*^

*** Correspondence:**Corresponding Author: tola@plen.ku.dk

# Supplementary Figures


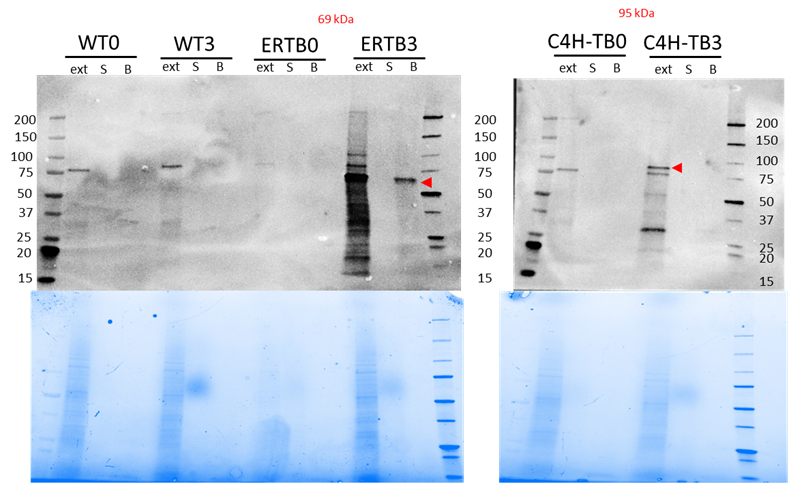


**Supplementary Figure 1**. Immunoblots to monitor affinity purification step. Protoplasts untransformed (WT) and transformed with plasmids for expression of ER-TurboID-EGFP (ERTB), C4H-TurboID (C4H-TB) and untransformed (WT) were incubated with 50 µM biotin for 0 and 3 hours. After several washing steps, protoplasts were lysed and total protein was isolated. Biotinylated proteins were affinity purified using streptavidin coated beads. **A)** Immunoblot of the control samples WT and ERTB at 0 and 3 hours after incubation. **B)** Immunoblot for samples expressing C4H-TurboID (C4H-TB). Lanes: ext: total protein extract, S: supernatant collected after affinity purification. B: proteins eluted from the beads after affinity purification. Red arrows indicate the band corresponding to “self” biotinylation.


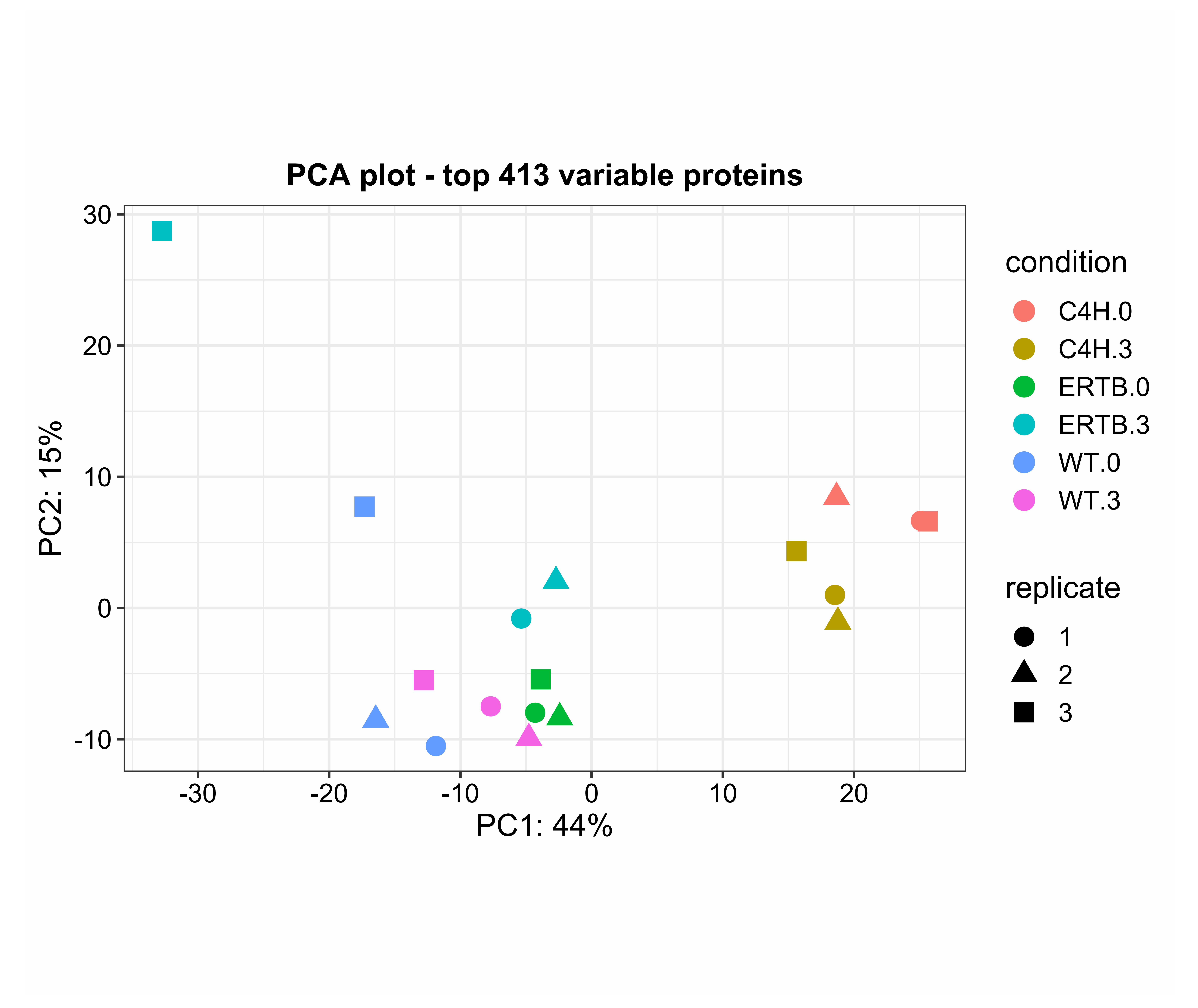


**Supplementary Figure 2.** Principal component analysis of proximity labeling experiment. Petunia petal protoplasts expressing C4Ha-TurboID (C4H), ER-TurboID (ERTB) and untransformed (WT) were collected at 0 and 180 min of biotin incubation. Each treatment is represented by a color, and different shapes correspond to each biological replicate.


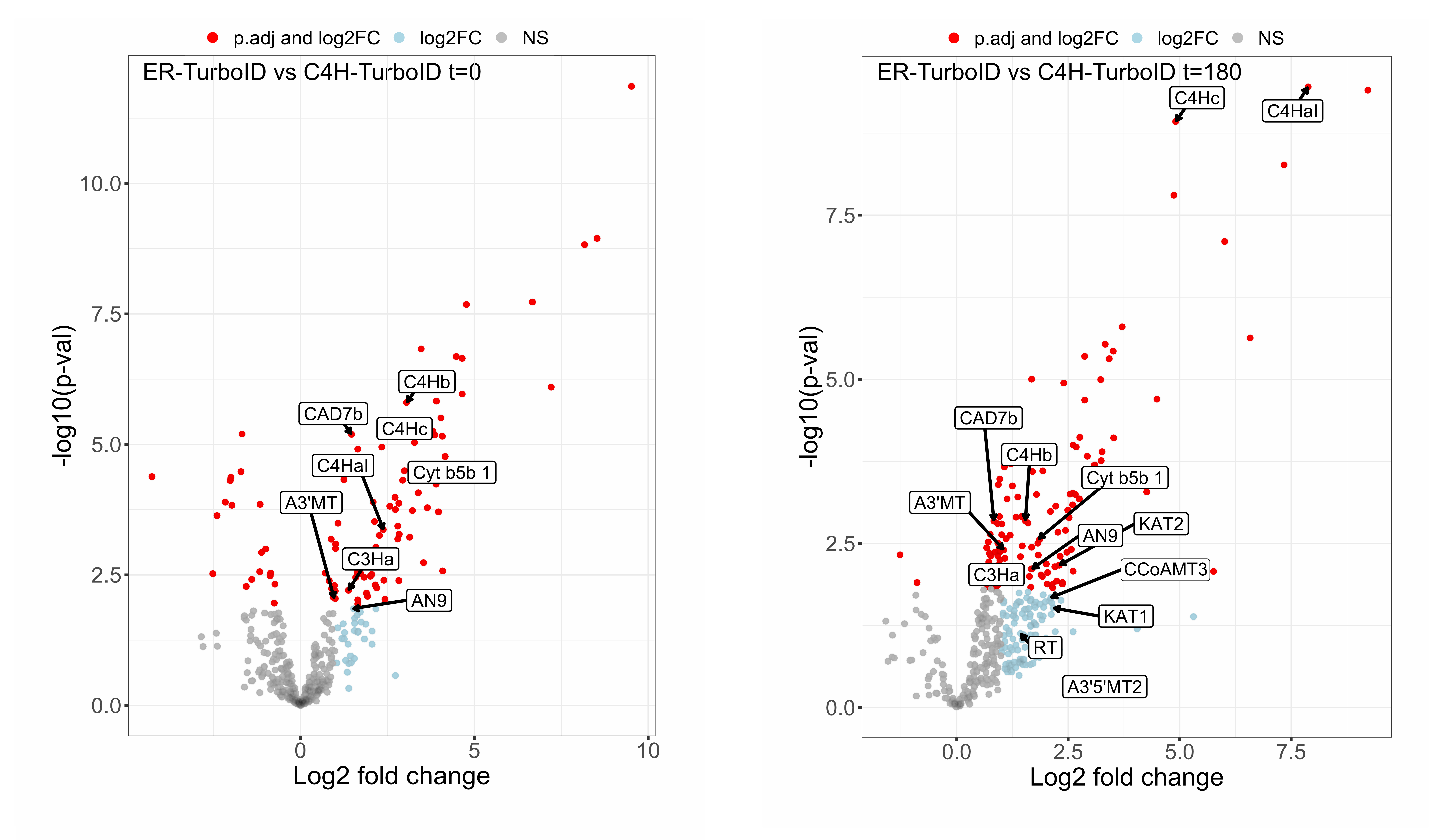


B

A

**Supplementary Figure 3.** Volcano plots of differentially enriched proteins in **A)** C4H-TurboID vs ER-TurboID-EGFP at t=0 and **B)** C4H-TurboID vs ER-TurboID-EGFP at t=180 min. Abbreviated enzyme names are indicated with boxes: A3’MT: Anthocyanin 3’-methyltransferase (MT/A3'MT); A3’5’MT2: Anthocyanin 3’5’-methyltransferase (MF2/A3'5’MT2); AN9: glutathione transferase; C4HI, C4Hb, C4Hc: C4H paralogs; C3Ha: cinnamate 3-hydroxylase a; CAD7: cinnamyl alcohol dehydrogenase 7a; CCoAMT3: Caffeoyl-CoA O-methyltransferase 3; Cyt b5b1: Cytochrome b5b 1; KAT1: 3-ketoacyl-CoA thiolase 1; KAT2: 3-ketoacyl-CoA thiolase 2; RT: anthocyanin rhamnosyltransferase.


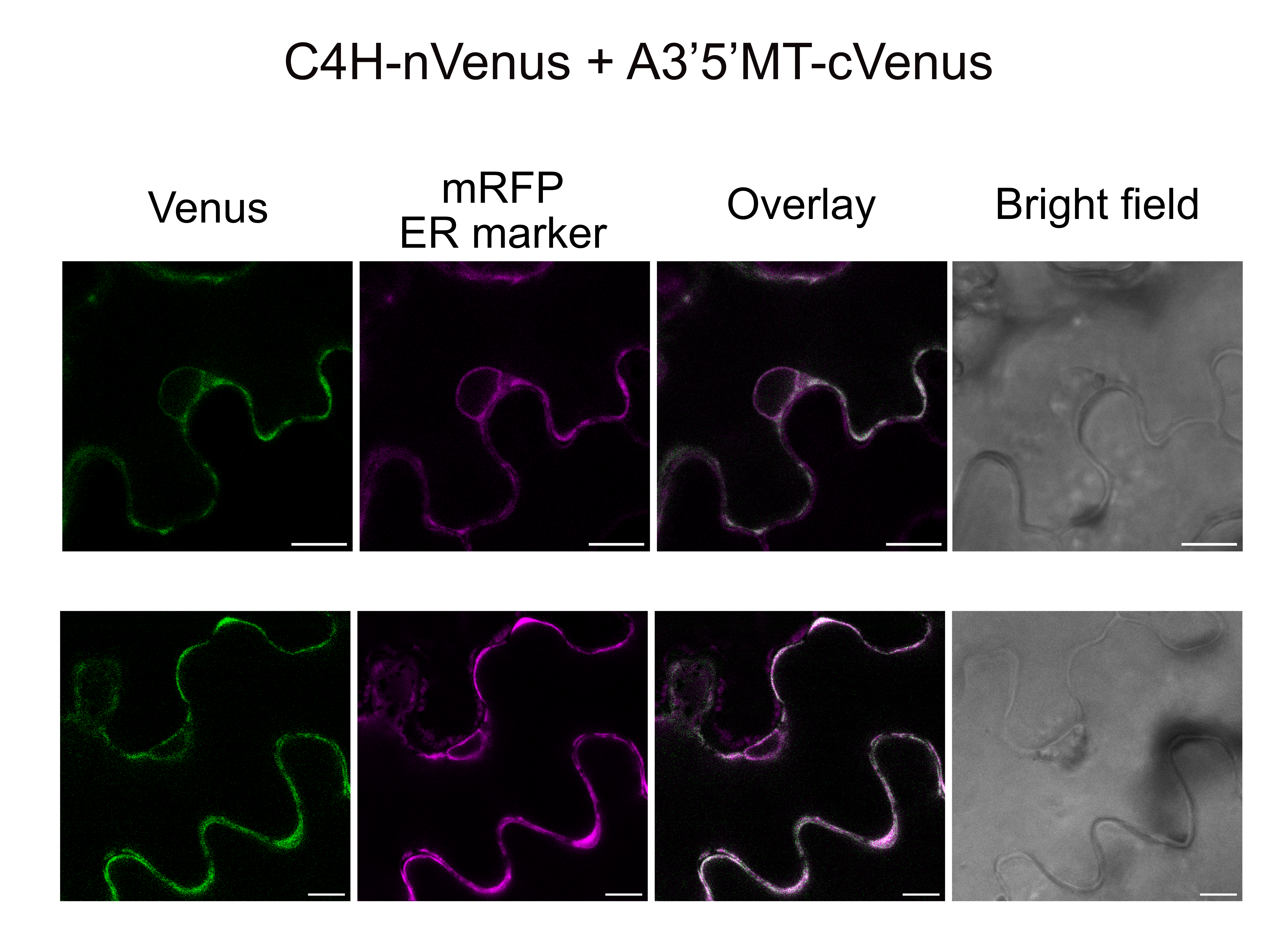


**Supplementary Figure 4.** Co-expression of C4H-nVenus and A3’5’MT-cVenus in *N. benthamiana* epidermal cells results in Venus fluorescence complementation, which co-localizes with ER marker (mRFP). Venus fluorescence is particularly faint in the ER portion surrounding the nucleus. Scale bars = 10 µm

**
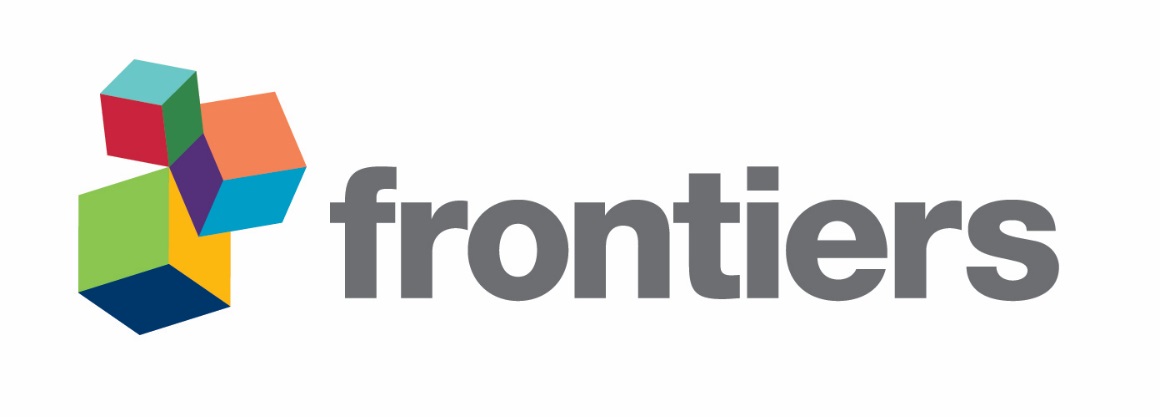
**
